# Unintended consequences and challenges of quality measurements in dentistry

**DOI:** 10.1186/s12903-019-0726-4

**Published:** 2019-03-01

**Authors:** Enihomo M. Obadan-Udoh, Jean M. Calvo, Sapna Panwar, Kristen Simmons, Joel M. White, Muhammad F. Walji, Elsbeth Kalenderian

**Affiliations:** 1 Department of Preventive and Restorative Dental Sciences, School of Dentistry, University of California San Francisco, 707 Parnassus Avenue, San Francisco, CA 94143 USA; 2 Pediatric Dentistry Post-Graduate Program, School of Dentistry, University of California San Francisco, 707 Parnassus Ave, San Francisco, CA 94143 USA; 3 Skourtes Institute, 6950 NE Campus Way, Hillsboro, OR 97124 USA; 4 Department of Diagnostic and Biomedical Sciences, School of Dentistry, University of Texas Health Science Center, 7500 Cambridge St., Houston, 77054 TX USA

**Keywords:** Ethics, Quality, Dentistry, Quality measures

## Abstract

**Background:**

In recent years, several state dental programs, researchers and the Dental Quality Alliance (DQA) have sought to develop baseline quality measures for dentistry as a way to improve health outcomes, reduce costs and enhance patient experiences. Some of these measures have been tested and validated for various population groups. However, there are some unintended consequences and challenges with quality measurement in dentistry as observed from our previous work on refining and transforming dental quality measures into e-measures.

**Main body:**

Some examples of the unintended consequences and challenges associated with implementing dental quality measures include: a de-emphasis on patient-centeredness with process-based quality measures, an incentivization of unethical behavior due to fee-for-service reimbursement systems, the risk of compromising patient and provider autonomy with plan-level measures, a disproportionate benefits of dental quality measurement going toward payers, and the risk of alienating smaller dental offices due to the resource-intensive nature of quality measurement.

**Conclusion:**

As our medical counterparts have embraced quality measurement for improved health outcomes, so too must the dental profession. Our ultimate goal is to ensure the delivery of high quality, patient-centered dental care and effective quality measurement is the first step. By continuously monitoring the performance of dental quality measures and their continued refinement when unintended consequences are observed, we can improve patient and population health outcomes.

## Background

From the early debates of an elitist profession bewildered by the advent of quality assurance and the reluctance to subject one’s practices to external scrutiny [1, 2] to the widespread prevalence of accreditation standards that serve as prerequisites for acceptance into the dental profession, [3–6] quality in dentistry has come a long way since its inception. While quality assurance is essential for ensuring the efficacy and effectiveness of dental interventions, it lacks the holistic and systems level focus that encourages continuous learning from engendering small changes to creating lasting solutions [7]. Quality improvement (QI), made popular by the Institute of Healthcare Improvement (IHI) through initiatives such as the Triple Aim [8], and furthered through efforts by the Joint Commission, has become the hallmark of forward-leaning healthcare institutions and learning healthcare systems [9, 10]. One crucial aspect of QI is the utilization of standardized measures of structure, process, and outcomes to assess performance and evaluate system changes [10, 11]. For the dental profession to keep pace with the healthcare system in the United States, there must be a shift from dentistry’s traditional understanding of quality systems encompassing risk management, quality control and quality assurance, to continuous QI through standardized measurement [12–14].

In recent years, several state dental programs, researchers and the DQA - *a team of dental stakeholders representing payers, educators, professional organizations, federal agencies, providers and the public*, have sought to develop baseline quality measures for dentistry as a way to improve health outcomes, reduce costs and enhance patient experiences [15–17]. Some of these measures (*n* = 18) have been tested and validated for various population groups (e.g. DQA Starter Set of Pediatric Oral Health Performance Measures) [18, 19] and nine of them have been endorsed by the National Quality Forum [20]. With the exception of some e-measures, all of these measures are derived from the administrative or claims-based data of public or private dental insurance agencies across the United States. As an integral part of the healthcare delivery system, and since oral health is essential to overall health [21], dental providers must enthusiastically embrace and support efforts to implement quality measures in the dental office [22]. This is our true north if we are to move towards achieving the six dimensions of quality - *safety, timeliness, efficiency, effectiveness, efficacy and patient-centeredness,* described by the Health and Medicine Division [*previously the Institute of Medicine (IOM)*] of the National Academy of Sciences, Engineering and Medicine (NASEM) [23].

## Main text

### What are some of the unintended consequences and challenges with quality measurement?

Our research team, through grant funding from the National Institute of Dental and Craniofacial Research (NIDCR) grant number 1R01DE024166, has been working to refine and transform dental quality measures into e-measures that are diagnosis-centered and can be easily deployed through the electronic health records (EHRs) at dental offices [24, 25]. Through this work, we have observed some unintended consequences and challenges with quality measurement in dentistry. These observations deserve attention to safeguard this nascent quality measurement effort in dentistry from avoidable pitfalls experienced by our medical counterparts [26]. The continuous monitoring of the performance of dental quality measures and their continuous refinement when unintended consequences are observed is essential to ensuring the sustenance of these efforts and that they truly reflect the quality of care being delivered. Some of these observations include:

### Process-based dental quality measures De-emphasize patient-centeredness

As described above, a critical hallmark of quality care is patient-centeredness. An improvement in the patient’s oral health outcome should always drive the dental care delivery process [27]. Crucial to achieving this goal is arriving at an accurate diagnosis and creating a treatment plan that matches this diagnosis in the appropriate sequence. The widely recognized NASEM definition of quality emphasizes outcomes-based quality measures, defining quality as “the degree to which health services for individuals and populations increases the likelihood of desired health outcomes and are consistent with current professional knowledge” [23]. However, evaluating treatment outcomes remains a challenge for the dental profession due to the slow-paced spread and implementation of the American National Standards Institute (ANSI)-approved standardized dental diagnostic terminology of the Systematized Nomenclature for Dental Diagnostic System (SNODDS) [28]. When procedure-based process measures are used as indicators of quality (as is the case with most prevailing dental quality measures), it becomes difficult to track the attainment of desired oral health outcomes (e.g. disease-free mouth, improved well-being) following procedure completion, or to ascertain the validity of diagnosis-procedure code pairs and their consistency with current scientific evidence. While process-based quality measures provide valuable insight into the standards of dental care delivery, they might also misrepresent the true quality of care being rendered [29–31]. Furthermore, it inadvertently promotes a culture that is intervention-prone rather than patient-centered, outcomes-focused and prevention-prone.

### Fee-for-service dental reimbursement systems incentivize unethical behavior

**Fig. 1:**
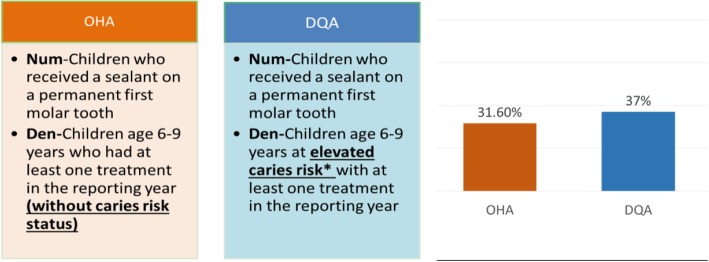
Sample Comparison of the Same Sealant Measures Dataset: Oregon Health Authority and DQA. The DQA measure reflects a higher quality score when only patients with elevated caries risk are given sealants

In tandem with the procedure-based process measures is the predominant use of fee-for-service payment mechanisms in dentistry [32]. The lack of a mandatory requirement for the use of diagnostic codes when processing billing claims means that dental providers are incentivized to simply complete a procedure irrespective of its indication. This has a dissuading effect on providers who deliver high quality and indicated care, howbeit ‘low-volume’ [17]. For example, providers obtaining a high-performance score for the proportion of children receiving sealants within their dental practice, although the sealants needed to be replaced every year due to low quality, or for the placement of sealants in low risk patients (see Fig. 1) [33–35]. While some promising payment mechanisms have been tested by our medical counterparts, such as ‘Pay-for-performance’ (P4P) or ‘value-based payments’, which provide financial incentives to clinicians and heath care providers for delivering high-quality care and an improvement in patient outcomes [36–38], they have not made their way into mainstream dentistry. Furthermore, unintended consequences have also been observed in P4P programs including the phenomenon of ‘gaming’, where providers “cherry pick” only patients who are expected to have better outcomes and exclude the ones expected to have poor outcomes in order to receive higher compensations ([39],para. 2). In dentistry, this may lead to dentists preferentially treating low-risk patients or those in need of less complex procedures [40]. If the dental profession is to tie reimbursement to performance, there needs to be a valid mechanism for adjusting for the case-mix and/or the severity of patients’ presenting conditions. Unfortunately, measures to assess case severity are not widely available or standardized in dentistry; therefore, more research will be needed before performance-based incentives can be implemented. The high percentage of out-of-pocket payments and multiple insurers per dental practice also means that the enforcement of P4P programs in dentistry will be an uphill battle [41].

### Plan-level measures risk compromising provider and patient autonomy

In recent years, there has been an increase in the emphasis on patient and family engagement in the healthcare delivery process. Providers are encouraged to work with patients as partners to ensure the delivery of high quality care. In fact, patients who are ‘activated’ have been shown to have better outcomes than those who are not [42]. Increased patient engagement and satisfaction also lead to increased provider satisfaction [43, 44]. However, the satisfaction of patients’ needs or preferences may require the performance of procedures that are neither conventional nor routine and may not be covered by the patient’s dental plan. While providers are focused on getting the best treatment outcomes for their patients, dental plans might be more interested in cost-savings and getting the most ‘bang for their buck’. The reliance on plan-level dental quality measures might mean that these providers would appear as outliers amongst their peers and be rated poorly for not adhering to conventional practices. When providers are faced with the choice of meeting their professional obligations to their patients or being truthful to the dental plans, some have chosen to ‘game the system’ by wrongly coding the procedures performed on their billing forms, as a way around this conflict. Conversely, other providers have chosen to perform procedures that do not meet the patient’s needs and even risk poor treatment outcomes just to comply with the plan-approved treatment recommendations [45, 46]. In order to avoid the unintended consequence of jeopardizing the provider-patient decision-making process, plan-level quality measures need to be interpreted with these nuances in mind [47].

### The benefits of dental quality measurement are skewed towards dental payment organizations

The implementation of quality measures by public and private dental payment organizations provide useful information to the payers regarding the performance of enrolled providers on a spectrum and allows them to identify outliers, infer expected treatment outcomes and evaluate adherence to evidence-based practices. However, most dental patients are not provided with access to this information and providers do not typically receive feedback about their performance in relation to their counterparts except when extreme practices are observed. This reduces the learning opportunities available to providers and colors their perception of the merits of quality measurement, especially when it consumes time, resources, and is not tied to their reimbursement. Similarly, patients are unaware of the performance scores of their providers and are unable to make informed choices when selecting their primary providers. Public reporting of provider-level dental quality measures, as is the case with Medicare providers, needs to be encouraged as a way to drive better provider performance and provide patients with validated quality measures upon which to base their assessments of providers [48]. In the absence of these measures, dental patients have relied on commercial review websites, such as Yelp, to choose their dental providers, which may not necessarily be a true reflection of the quality of care provided in terms of treatment outcomes [49, 50] but rather a reflection on the provider’s chair-side manners [43, 51, 52]. To develop provider-level measures that assess treatment outcomes, more funding is needed for large, observational research studies, and the creation of centralized, publicly accessible reporting systems [29].

Although there is a shifting trend towards Dental Service Organizations (DSOs) and corporate dental chains, the majority of dental offices in the United States are still solo-practices or small group practices [36, 53, 54]. This often means limited staffing and the absence of dedicated staff to handle back-office operations such as implementing health information technology (IT), performing chart review audits, data entry, and organizing quality improvement activities. Any quality measurement attempts at these smaller offices will come at the expense of productivity and chairside time. While the benefits and potential savings from delivering high quality care range from improved efficiency and wastage elimination, to better patient and provider satisfaction, the upfront costs are often untenable for a majority of these solo practices. As we develop quality measures in dentistry, significant attention needs to be focused on developing e-measures that can be easily deployed through EHRs with minimal staff effort and time. Pulling structured data from the EHRs also has the added advantage of reducing documentation fatigue that comes with having to complete redundant forms that have no bearing on the care being provided just to meet certain quality metrics [55]. Furthermore, it has been shown that dentists are less likely to participate in programs that require extensive or complicated documentation completion [56]. The implementation of quality measures without the consideration of the time and financial implications to providers may ultimately lead to low provider engagement in quality measurement or a reduction in time spent providing face-to-face patient-centered care.

## Conclusions

As our medical counterparts have embraced quality measurement for improved patient and population health outcomes, so too must the dental profession. The standardization and implementation of diagnostic terminologies in dental offices nationwide is an important step towards achieving widespread quality measurement [57–59]. Without diagnostic terms, the dental profession is severely limited in its ability to measure appropriate treatment and health outcomes. Furthermore, as quality measurement evolves, the challenge of the dental reimbursement structure and payment mechanisms cannot be ignored. It is essential that new dental quality measures account for the subtle nuances involved with delivering high quality dental care, and that smaller dental offices or solo practitioners are not left behind. In the end, our ultimate goal is to ensure the delivery of high quality, patient-centered dental care and effective quality measurement is the first step.

## Abbreviations

ANSI: American National Standards Institute
CDT: Current Dental Terminology
DEN: Denominator
DQA: Dental Quality Alliance
DSOs: Dental service organizations
EHRs: Electronic health records
IHI: Institute of Healthcare Improvement
IOM: Institute of Medicine
IT: Information technology
NASEM: National Academy of Sciences, Engineering and Medicine
NIDCR: National Institute of Dental and Craniofacial Research
NUM: Numerator
OHA: Oregon Health Authority
P4P: Pay-for-performance
QI: Quality improvement
SNODDS: Systematized Nomenclature for Dental Diagnostic System
